# Robust performance of deep learning for distinguishing glioblastoma from single brain metastasis using radiomic features: model development and validation

**DOI:** 10.1038/s41598-020-68980-6

**Published:** 2020-07-21

**Authors:** Sohi Bae, Chansik An, Sung Soo Ahn, Hwiyoung Kim, Kyunghwa Han, Sang Wook Kim, Ji Eun Park, Ho Sung Kim, Seung-Koo Lee

**Affiliations:** 1 Department of Radiology, National Health Insurance Service Ilsan Hospital, Goyang, 10444 Korea; 2 Research and Analysis Team, National Health Insurance Service Ilsan Hospital, Goyang, 10444 Korea; 3 Department of Radiology, Severance Hospital, Research Institute of Radiological Science and Center for Clinical Image Data Science, Yonsei University College of Medicine, 50-1 Yonsei-ro, Seodaemun-gu, Seoul, 03722 Korea; 4 School of Biomedical Engineering, Korea University College of Health Science, Seoul, 02841 Korea; 5 Department of Radiology and Research Institute of Radiology, Asan Medical Center, University of Ulsan College of Medicine, Seoul, 05505 Korea

**Keywords:** Cancer, Computational biology and bioinformatics, Diseases, Medical research, Oncology, Mathematics and computing

## Abstract

We evaluated the diagnostic performance and generalizability of traditional machine learning and deep learning models for distinguishing glioblastoma from single brain metastasis using radiomics. The training and external validation cohorts comprised 166 (109 glioblastomas and 57 metastases) and 82 (50 glioblastomas and 32 metastases) patients, respectively. Two-hundred-and-sixty-five radiomic features were extracted from semiautomatically segmented regions on contrast-enhancing and peritumoral T2 hyperintense masks and used as input data. For each of a deep neural network (DNN) and seven traditional machine learning classifiers combined with one of five feature selection methods, hyperparameters were optimized through tenfold cross-validation in the training cohort. The diagnostic performance of the optimized models and two neuroradiologists was tested in the validation cohort for distinguishing glioblastoma from metastasis. In the external validation, DNN showed the highest diagnostic performance, with an area under receiver operating characteristic curve (AUC), sensitivity, specificity, and accuracy of 0.956 (95% confidence interval [CI], 0.918–0.990), 90.6% (95% CI, 80.5–100), 88.0% (95% CI, 79.0–97.0), and 89.0% (95% CI, 82.3–95.8), respectively, compared to the best-performing traditional machine learning model (adaptive boosting combined with tree-based feature selection; AUC, 0.890 (95% CI, 0.823–0.947)) and human readers (AUC, 0.774 [95% CI, 0.685–0.852] and 0.904 [95% CI, 0.852–0.951]). The results demonstrated deep learning using radiomic features can be useful for distinguishing glioblastoma from metastasis with good generalizability.

## Introduction

Glioblastoma and brain metastasis are the most common malignant brain tumors in adults^1^. Differentiation of these two types of tumors is important for planning further diagnostic workup and treatment^2^. In case of a suspected brain metastasis, a full workup is required to identify the primary tumor and its dissemination status. Furthermore, treatment strategies differ for these tumors^3^; En bloc resection is preferred for metastasis and stereotactic radiosurgery can be performed for metastases less than 3–4 cm^4^, whereas maximal safe resection followed by molecular classification and concurrent chemoradiotherapy should be considered for glioblastomas^5^.

Currently, definitive diagnosis of glioblastoma or metastasis is based on histopathology. However, this poses the risk of morbidity and mortality^6^, particularly in cases of tumors located near eloquent areas or in patients with advanced age. Therefore, accurate non-invasive radiologic diagnosis would be of great use.

Multiplicity of lesions, a cerebellar location, and a clinical history of systemic cancer favor the diagnosis of brain metastasis over glioblastoma in patients with enhancing brain masses^7,8^. However, half of brain metastases present as a single lesion^9^ and approximately 15–30% of brain metastases present as an initial manifestation of systemic malignancy^10,11^, which may cause diagnostic difficulties. In addition, the ability to differentiate glioblastoma from a single brain metastasis on conventional magnetic resonance (MR) imaging alone remains challenging because of their similar appearance on imaging; both can present with ring-enhancement, intratumoral necrosis, and peritumoral T2 hyperintensity^12,13^. Although advanced MR techniques such as MR spectroscopy^14,15^, perfusion MR imaging^12,16,17^, and diffusion-weighted imaging (DWI)^17,18^ have demonstrated various degrees of success in distinguishing metastasis from glioblastoma, the validity of their roles in the differentiation of these tumors is insufficient^19^.

Radiomics, high-throughput mining of quantitative imaging features, has the potential to provide information about the underlying pathophysiology that is difficult to discover by visual inspection^20^. Several previous studies have shown that radiomics combined with traditional machine learning is capable of discriminating glioblastoma from lymphoma^21,22^, predicting genetic information about the tumor^23,24^, and predicting the treatment response^25^ or survival^26–28^.

Deep learning is a subset of machine learning consisting of computational units of multiple layers resembling the multilayered human cognition system^29^. There have been several studies using deep learning to predict glioma grading^30^, glioma genetic mutation^31^ or survival^32^. Recently, it was also demonstrated that the application of deep learning to radiomics could successfully discriminate glioblastoma from lymphoma^33^.

Therefore, we evaluated the diagnostic performance and generalizability of radiomics-based machine learning models including deep learning using deep neural network (DNN) for discriminating between glioblastoma and single brain metastasis.

## Results

### Clinical characteristics of patients

**Figure 1:**
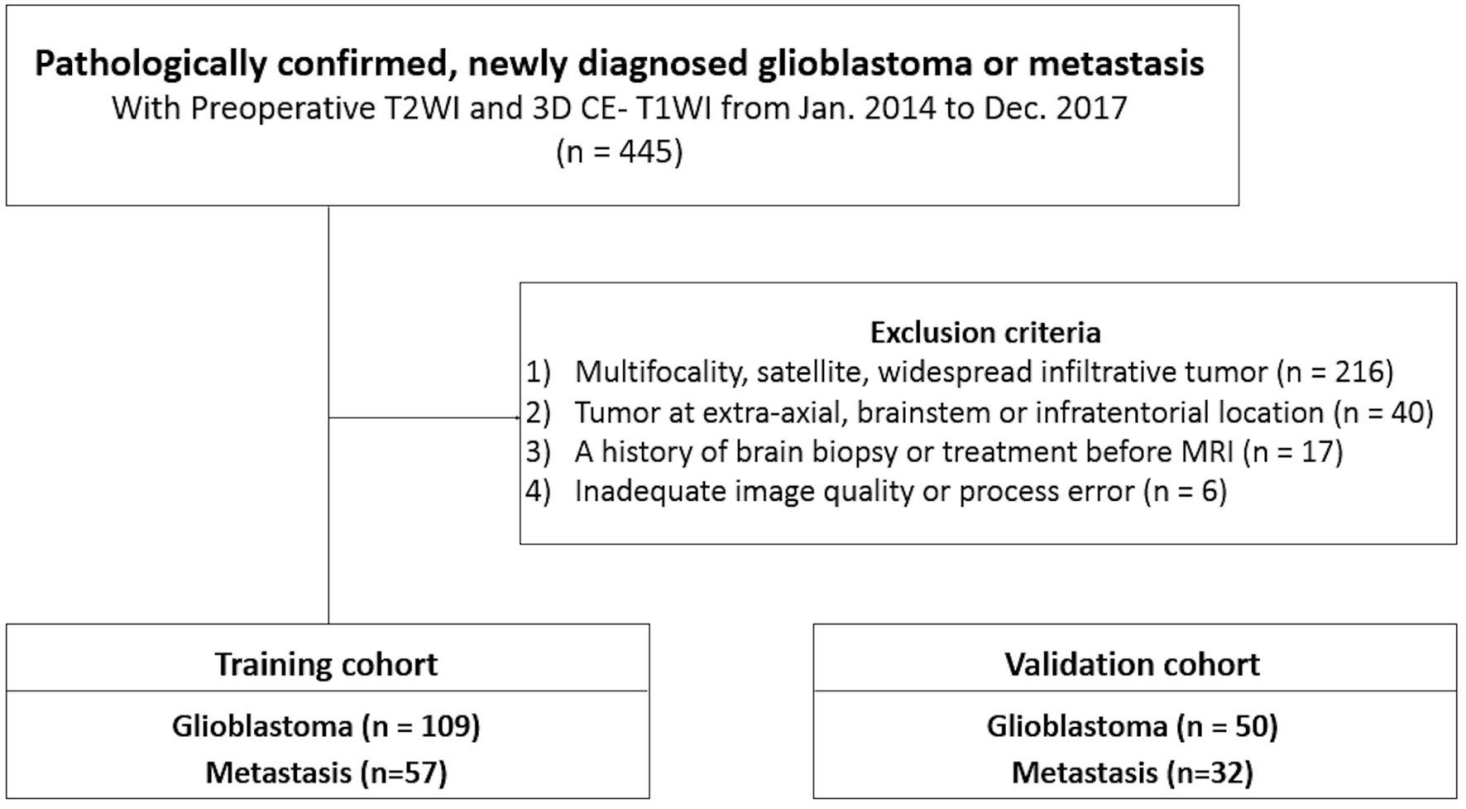
Flow chart of patient inclusion. *T2WI* T2-weighted image, *3D CE-T1WI* three-dimensional contrast-enhanced T1-weighted image, *IDH* isocitrate dehydrogenase.

The baseline demographics and clinical characteristics of the patients are summarized in Table 1. One-hundred-and-sixty-six patients (109 glioblastomas and 57 metastases) (Fig. 1) in the training cohort had a mean age of 59 years ± 13 (standard deviation) (range, 18–83 years), with 109 men and 57 women. Eighty-two patients (50 glioblastomas and 32 metastases) in the external validation cohort had a mean age of 59 years ± 13 (range, 18–82 years), with 48 men and 34 women. The most common primary cancer of metastasis was lung cancer, followed by breast cancer and colorectal cancer, in both cohorts without significant difference in proportion (*P* = 0.818). Among the patients with brain metastasis, 50 of 57 patients (87.7%) in the training cohort and 16 of 32 patients (50%) in the validation cohort had known primary cancer at the time of diagnosis of brain metastasis. There was no significant difference in age between patients with glioblastoma and metastasis in either the training or the validation cohort. The mean interval between MR imaging and pathological confirmation was 10.9 days ± 17.2 (standard deviation) in glioblastoma patients and 33.5 days ± 79.6 in metastasis patients.

**Table 1:** Baseline demographics and clinical characteristics of patients.

	Training cohort (n = 166)			External validation cohort (n = 82)		
	Glioblastoma (n = 109)	Metastasis (n = 57)	*P* value	Glioblastoma (n = 50)	Metastasis (n = 32)	*P* value
Age (years)*	58.4 ± 13.6	58.4 ± 12.1	0.990	58.3 ± 13.7	60.7 ± 11.0	0.411
Male	78 (71.6%)	31 (54.4%)	0.027	30 (60.0%)	18 (56.3%)	0.737
*Primary site of metastasis*						
Lung		24 (42.1%)			15 (46.9%)	
Breast		6 (10.5%)			4 (12.5%)	
Colon/rectum		6 (10.5%)			4 (12.5%)	
Urinary tract		5 (8.8%)			3 (9.3%)	
Genitourinary tract		10 (17.5%)			6 (18.8%)	
Others		11 (19.3%)			3 (9.3%)	

*Data are means ±  SD.

### Traditional machine learning

**Figure 2:**
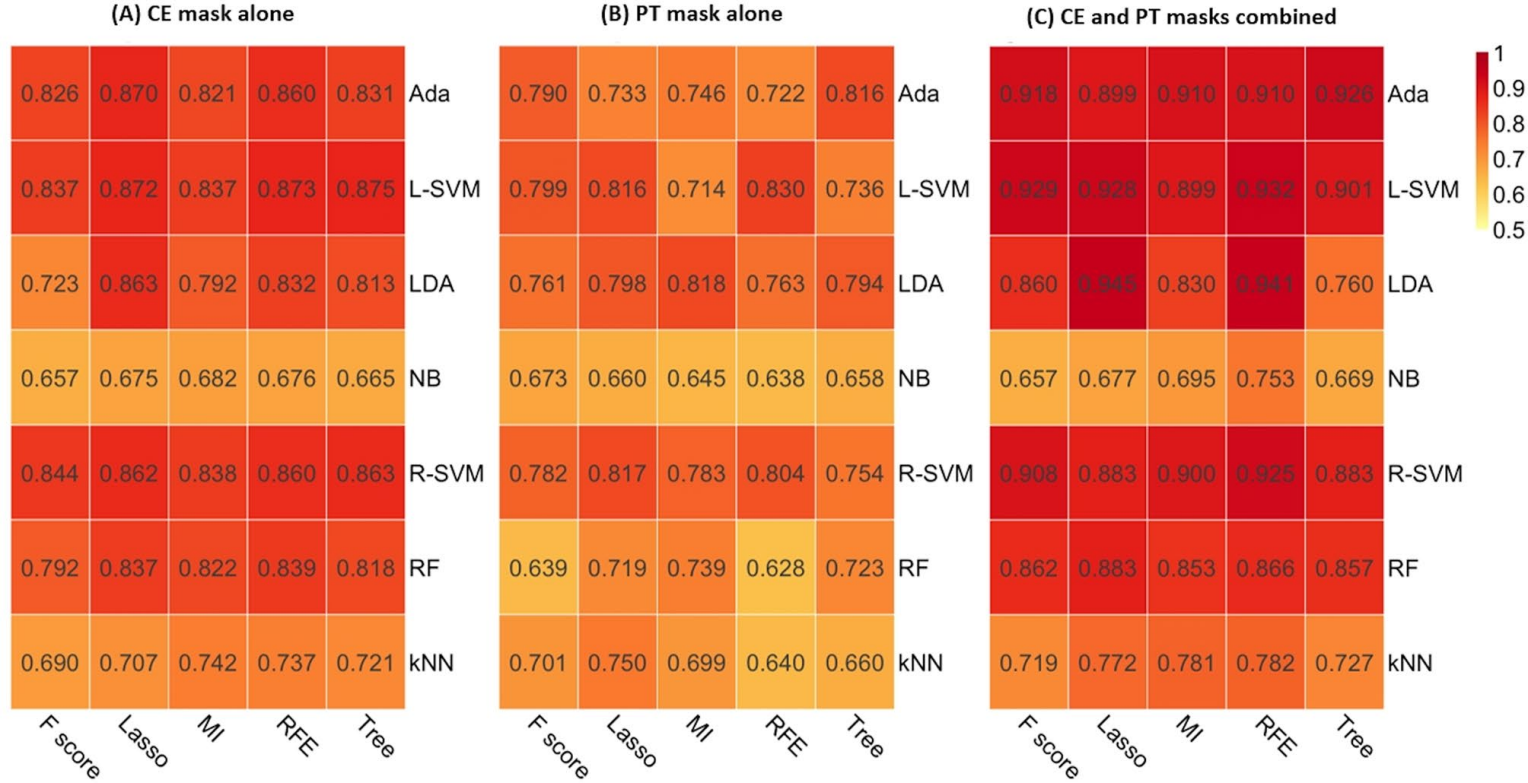
Heatmap depicting the mean area under the receiver operating characteristic curve of five machine learning classifier (columns) and seven feature selection (rows) methods in the training set. (**A**) CE mask alone, (**B**) PT mask alone, (**C**) CE and PT masks combined. *CE* contrast-enhancing, *PT* peritumoral T2 hyperintense, *MI* mutual information,* RFE*  recursive feature elimination, *Lasso* least absolute shrinkage and selection operator, *kNN* k-nearest neighbor, *NB* naïve Bayes, *RF* random forest, *Ada* adaptive boosting, *L-SVM* linear support vector machine, *R-SVM* radial basis function support vector machine, *LDA* linear discriminant analysis.

Two-hundred-and-sixty-five radiomic features extracted from contrast-enhancing (CE) mask (defined as contrast-enhancing areas of tumor) or peritumoral T2 hyperintense (PT) mask (defined as non-enhancing T2 hyperintense areas excluding contrast-enhancing or necrotic portions) were used as input data. Among machine learning classifiers, adaptive boosting (AdaBoost), support vector machine using linear kernel (L-SVM), and linear discriminant analysis (LDA) were consistently the best three models in the cross validation, irrespective of the image mask used (Table 2 and Supplementary Tables S1–S3). The performances of all the combinations of feature selection method and classifier according to the mask used in the training cohort are summarized in Fig. 2 and Supplementary Tables S1–S3. In the external validation, the best performance was achieved by AdaBoost combined with tree-based feature selection when both CE and PT masks were used, with an area under receiver operating characteristic curve (AUC) of 0.890 (95% confidence interval [CI], 0.823–0.947), a sensitivity of 80.0% (95% CI, 62.3–90.0), a specificity of 87.5% (95% CI, 71.0–94.5), and an accuracy of 82.9% (95% CI, 73.0–90.3), respectively, for distinguishing glioblastoma from metastasis. The diagnostic performances of the three machine learning classifiers according to the mask are summarized in Table 2.

**Table 2:** Diagnostic performance of traditional machine learning, deep learning, and human readers.

Data	Classifier	Cross validation	External validation			
		Mean AUC	AUC	Sensitivity (%)	Specificity (%)	Accuracy (%)
*Traditional machine learning*						
CE mask	AdaBoost with LASSO	0.870	0.858 (0.787–0.926)	68.0 (53.3–80.5)	93.8 (79.2–99.2)	78.0 (67.5–86.4)
	L-SVM with Tree-based selection	0.875	0.833 (0.755–0.904)	62.0 (47.2–75.4)	93.8 (79.2–99.2)	74.4 (63.6–83.4)
	LDA with LASSO	0.863	0.818 (0.737–0.891)	64.0 (49.2–77.1)	87.5 (71.0–96.5)	73.2 (62.2–82.4)
PT mask	AdaBoost with Tree-based selection	0.816	0.773 (0.668–0.870)	94.0 (83.5–98.8)	34.4 (18.6–53.2)	70.7 (59.7–80.3)
	L-SVM with RFE	0.830	0.803 (0.718–0.879)	86.0 (75.5–96.5)	65.6 (46.8–81.4)	78.0 (67.2–88.8)
	LDA with MI	0.818	0.787 (0.695–0.870)	94.0 (83.5–98.8))	50.0 (31.9–68.1	76.8 (66.2–85.4)
Combined mask	AdaBoost with Tree-based selection	0.926	0.890 (0.823–0.947)	80.0 (62.3–90.0)	87.5 (71.0–94.5)	82.9 (73.0–90.3)
	L-SVM with RFE	0.932	0.886 (0.798–0.927)	80.0 (62.3–90.0)	84.4 (67.2–94.7)	81.7 (71.6–89.4)
	LDA with LASSO	0.945	0.899 (0.839–0.951)	84.0 (70.9–92.8)	78.1 (70.9–90.7)	81.7 (71.6–89.4)
*Deep learning*						
CE mask	DNN	0.887	0.887 (0.812–0.951)	62.5 (45.7–79.3)	96.0 (90.6–100)	82.9 (74.8–91.1)
PT mask	DNN	0.865	0.825 (0.722–0.887)	75.0 (60.2–90.1)	82.0 (71.4–92.6)	79.3 (70.5–88.0)
Combined mask	DNN	0.986	0.956 (0.918–0.990)	90.6 (80.5–100)	88.0 (79.0–97.0)	89.0 (82.3–95.8)
*Human reading*						
Images	Reader 1		0.774 (0.685–0.852)	97.0 (91.1–100)	50.0 (36.1–63.9)	68.7 (58.7–78.7)
Images	Reader 2		0.904 (0.852–0.951)	81.8 (68.7–95.0)	78.0 (66.5–89.5)	79.5 (70.8–88.2)

Values in parentheses are 95% confidence intervals.

*AUC* area under the receiver operating characteristic curve, *CE* contrast-enhancing, *PT* peritumoral T2 hyperintense, *AdaBoost* adaptive boosting, *L-SVM* linear support vector machine, *LDA* linear discriminant analysis, *RFE* recursive feature elimination, *DNN* deep neural net.

### Feature importance and stability

The permutation feature importance is listed in descending order of total score in Table 3 (top 10 features used by the three best models: AdaBoost, L-SVM, and LDA) and Supplementary Table S4 (all features with a total score > 0 for all models). Overall, the most important radiomic feature was first-order median and 10 percentile values assessed on T2WI.

**Table 3:** Importance score and stability of top 10 radiomic features.

Radiomic feature	Permutation feature importance				ICC (95% CI)
	AdaBoost	L-SVM	LDA	Total	
First order, median (CE mask on T2WI)	15.8	100.0	45.9	161.7	0.989 (0.968–0.996)
First order, 10 percentile (CE mask on T2WI)	86.8	55.3	7.7	149.8	0.942 (0.844–0.979)
GLCM, inverse difference (CE mask on CE-T1WI)	31.6	1.3	100.0	132.9	0.995 (0.985–0.998)
Shape, maximum 2D diameter row (PT mask on T2WI)	78.9	43.4	9.0	131.4	0.969 (0.915–0.989)
First order, median (PT mask on T2WI)	60.5	65.8	NS	126.3	0.863 (0.660–0.950)
GLRLM, low gray level run emphasis (CE mask on CE-T1WI)	0.0	44.7	63.4	108.1	0.995 (0.985–0.998)
Shape, flatness (PT mask on T2WI)	60.5	34.2	5.5	100.2	0.866 (0.665–0.951)
GLSZM, gray level non uniformity (CE mask on T2WI)	100.0	NS	NS	100.0	0.972 (0.921–0.990)
GLCM, inverse variance (CE mask on CE-T1WI)	0.0	36.8	57.7	94.5	0.994 (0.980–0.998)
GLCM, autocorrelation (CE mask on CE-T1WI)	0.0	0.0	94.3	94.3	0.991 (0.972–0.997)

Importance scores of all the radiomic features used for seven machine learning classifiers are presented in Supplementary Table S4.

*AdaBoost* adaptive boosting, *L-SVM* linear support vector machine, *LDA* linear discriminant analysis, *ICC* intraclass correlation coefficient, *CI* confidence interval, *CE* contrast-enhancing, *T2WI* T2-weighted imaging, *T1WI* T1-weighted imaging, *PT* peritumoral T2 hyperintense, *GLCM* gray level co-occurrence matrix, *GLRLM* gray level run length matrix, *GLSZM* gray level size zone matrix, *NS* not selected for this classifier.

Of 265 radiomic features, 197 (74.3%), 25 (9.4%), 22 (8.3%), and 21 (7.9%) showed excellent, good, moderate, and poor feature stability, respectively. All the top 10 features showed good or excellent stability except for two features that showed moderate stability (Table 3).

### Deep learning

The DNN model showed higher diagnostic performance than the traditional machine learning models. In the cross validation, the mean AUCs were 0.887, 0.865, and 0.986 on CE mask alone, PT mask alone, and both masks, respectively. In the external validation, the optimized DNN showed the best performance when both masks were used, with the AUC, sensitivity, specificity, and accuracy of were 0.956 (95% CI, 0.918–0.990), 90.6% (95% CI, 80.5–100), 88.0% (95% CI, 79.0–97.0), and 89.0% (95% CI, 82.3–95.8), respectively (Table 2).

### Human reading

Human reader 1 (S.B., with 3 years’ experience in neuro-oncological imaging) obtained an AUC of 0.774 (95% CI, 0.686–0.852), a sensitivity of 50.0% (95% CI, 36.1–63.9%), a specificity of 97.0% (95% CI, 91.1–100%), and an accuracy of 68.7% (95% CI, 58.7–78.7%). Reader 2 (S.S.A, with 10 years’ experience) obtained an AUC of 0.904 (95% CI, 0.850–0.949), a sensitivity of 78.0% (95% CI, 66.5–89.5%), a specificity of 81.8% (95% CI, 68.7–95.0%), and an accuracy of 79.5% (95% CI, 70.8–88.2%) in the external validation cohort (Table 2).

### Inter-rater agreement

The overall agreement between AdaBoost, L-SVM, LDA, DNN, and two human readers was moderate with a multi-rater kappa value of 0.472. The agreement among the machine learning classifiers (i.e., AdaBoost, L-SVM, LDA, and DNN) was substantial with kappa values ranging from 0.607 to 0.742, whereas the agreement between the two human readers was moderate with a kappa of 0.505. The agreement between machine learning and human reading was slight to moderate with kappa values ranging from 0.17 to 0.546 (Supplementary Table S5).

## Discussion

We explored the diagnostic performance of radiomics using traditional machine learning or deep learning classifiers for differentiating glioblastoma from single brain metastasis. The deep learning algorithm (i.e., DNN) performed better than the best-performing traditional machine learning classifiers or human readers and demonstrated good generalizability in the external validation.

Previous studies that had conducted quantitative morphometric analysis revealed significant differences in tumor shape between glioblastoma and metastasis^13,34^. These reports explained that the aggressive proliferation and tendency of glioblastoma to grow in white matter regions, the cerebral spinal fluid, or meninges contribute to its irregular shape, whereas metastases have a relatively low proliferation rate and expand more homogeneously, with adhesion among cells, yielding a compact spheroidal tumor shape. Human readers can easily recognize and utilize the shape features, but many of the other radiomic features are not recognizable by humans. The fact that machine learning can make use of information from image data that humans are unable to use may partly explains the comparable or better diagnostic performances of machine learning as well as the lower agreements between machine learning and human readers in our study. In addition, our results confirm that the image interpretation by human readers may vary; in our study, their agreement was worse than those between machine learning classifiers. Robust and reproducible machine learning could help overcome possible human error and bias.

A recent study explored four machine learning algorithms using radiomic features to differentiate glioblastoma from brain metastasis, reporting that SVM showed good performance^35^. Compared to their study, we included only solitary and supratentorial tumors for which imaging diagnosis is challenging and used deep learning in addition to traditional machine learning. Our diagnostic models were validated in the external cohort to assure generalizability of the model across centers. Furthermore, we examined the importance of radiomic features used by machine learning algorithms.

The analysis of feature importance showed that three of the ten most important features were those assessed in peritumoral areas. The peritumoral areas of glioblastoma and metastasis are different in underlying pathophysiology. Quantitative MR techniques have shown the tumoral pattern of metabolite ratio, increased neovascularity, and increased cellularity in peritumoral area of glioblastoma^15,18^. Therefore, we hypothesized that radiomic features in the peritumoral areas contain unique information to complement those in the enhancing tumor itself for differentiating between glioblastoma and metastasis, which is supported by the results that the diagnostic performance was highest when both CE and PT masks were used regardless of the type of machine learning algorithm.

The good performance of DNN in our study may be attributed to that fact that DNN can adapt flexibly to a given task and data structure data. DNN was implemented using a multi-input model rather than a sequential model in this study. This non-sequential architecture may facilitate efficient and effective extraction of the relevant radiomic features provided by different sequences and masks. A naïve approach would be to train three separate sequential models and then obtain a weighted average of their predictions, but this approach would be suboptimal because the information extracted by the models may be redundant.

In this study, we applied DNN to handcrafted radiomic features, rather than using convolutional neural network (CNN)-based end-to-end deep learning. Although a CNN is generally superior to other types of DNN in visual classification, it is more susceptible to overfitting and requires a large amount of data for model training and parameter tuning^36^. A recent study for distinguishing glioblastoma from lymphoma found that CNN performed markedly worse than machine learning or DNN applied to radiomics in an external validation set (AUC, 0.486 vs. 0.811 vs. 0.947), even with data augmentation and the use of a pretrained network^33^. This may have been due to the limited size of the MR imaging dataset and overfitting, resulting in decreased performance in an independent validation dataset. A further study using large and heterogeneous datasets might overcome the overfitting problem and establish robust CNN algorithm to diagnose brain tumors.

Our prediction models were trained to diagnose single brain tumor (i.e., glioblastoma vs. metastasis). However, the pathologic and molecular typing of glioblastoma also plays an important role in the diagnosis as well as prediction of prognosis, still necessitating biopsy. As many recent studies have reported remarkable success of machine learning in brain tumor grading^30^, molecular classification^28,31^ and survival prediction^26,32^, a future study is warranted to develop a robust machine learning algorithm into which molecular subtyping and prognosis prediction are integrated.

## Limitations

This study had several limitations. First, tumors were semiautomatically segmented; this could be user-dependent, labor-intensive, and time-consuming. Previous studies have proposed deep learning-based tumor segmentation algorithms with robust performance^37,38^. Automated tumor segmentation, which could possibly be integrated into an automated processing pipeline, would allow automated and quantitative analysis of MR images, minimize subjectivity, and facilitate larger-scale studies in neuro-oncology^37^. Second, we did not include advanced sequences, such as DWI or perfusion MR imaging, in our analysis. Further multiparametric analysis would increase diagnostic performance in differentiating glioblastoma and metastasis. However, advanced techniques require additional scans and their protocols differ among institutions. In contrast, conventional MR imaging is commonly performed for patients with neurologic symptoms in clinical practice, and thus radiomics analysis of conventional MR imaging can be used more widely. Lastly, the MR images in our study were obtained using heterogeneous MR scanner types, with various acquisition parameters, which can affect radiomic features and quantitative analysis^39^, Nonetheless, the diagnostic performance of the radiomics model remained high in the validation cohort, which substantiates the good generalizability of the model.

## Conclusion

In conclusion, this study showed that deep learning based on radiomic features can be useful for discriminating between glioblastoma and single brain metastasis, with good generalizability across institutions.

## Methods

This retrospective study was approved by the Severance Hospital Institutional Review Board (No. 4-2019-1084), and the requirement for obtaining informed consent was waived.

### Study population

The records of 445 consecutive adult patients with a newly diagnosed glioblastoma or brain metastasis, who underwent preoperative MR imaging from January 2014 to December 2017 at a tertiary medical center were reviewed retrospectively. Patients were included on the basis of the following criteria: (a) pathologically confirmed glioblastoma or metastasis; (b) preoperative MR imaging, including T2-weighted imaging (T2WI) and three-dimensional (3D) contrast-enhanced T1-weighted imaging (CE-T1WI). The exclusion criteria were as follows: (a) multifocality, the presence of satellite tumors, or widespread infiltrative tumor involving three or more cerebral lobes (i.e., gliomatosis) (n = 216); (b) tumor at extra-axial, brainstem, or infratentorial location (n = 40); (c) a history of brain biopsy or treatment before MR imaging (n = 17); (d) inadequate image quality or process error (n = 6). Consequently, 166 consecutive patients (109 glioblastomas and 57 metastases) comprised the training cohort for model development (Fig. 1).

For external validation of the model, another independent cohort of 82 patients with pathologically confirmed glioblastoma (n = 50) or single metastasis (n = 32), identified at another tertiary medical center between January 2014 and December 2017, was used as the validation cohort. These patients satisfied the same inclusion and exclusion criteria and were randomly selected from an electronic database.

### MR data

MR studies of the training cohort were performed on one of three 3-T MR scanners (Achieva, Philips, Best, the Netherlands; Ingenia, Philips; Discovery MR750, GE, Milwaukee, WI) with an eight-channel sensitivity-encoding head coil. Achieva, Ingenia and Discovery MR750 were used for 74, 32 and 3 patients with glioblastoma, and 31, 15 and 11 patients with metastasis, respectively. MR scans of the external validation cohort were performed on a 3-T MR scanner (Achieva; Philips) with an eight-channel sensitivity-encoding head coil. Imaging protocols in both institutions included the following sequences: T2WI, T1WI, FLAIR imaging, and CE-T1WI. Detailed imaging parameters used in the two institutions are summarized in Supplementary Table S6.

### Image processing and radiomic feature extraction

The overall study pipeline is shown in Fig. 2. Tumor segmentation was performed on (a) CE lesions (defined as enhancing areas on CE-T1WI) and (b) PT lesions (defined as non-enhancing T2 hyperintense areas excluding contrast-enhancing or necrotic portions). Two regions-of-interest (ROIs) were drawn by semiautomated methods, including signal intensity thresholding, region growing, and edge detection with the Medical Image Processing, Analysis, and Visualization software package, Version 8.0 (National Institutes of Health; https://mipav.cit.nih.gov). ROIs were drawn by a neuroradiologist (S.B., with 3 years of experience in neuro-oncological imaging) and confirmed by another neuroradiologist (S.S.A., with 10 years of experience). Disagreements were resolved by discussion to consensus. Radiologists were blinded to the patients’ diagnosis.

3D CE-T1WI and T2WI were resampled into a uniform voxel size of 1 × 1 × 1 mm^3^ across all patients. 3D CE-T1WI were registered to T2WI. N4 bias-correction was performed to remove low-frequency intensity non-uniformity from images, using Advanced Normalization Tools (https://stnava.github.io/ANTs), and signal intensity was normalized by using the WhiteStripe R package^40^.

Radiomic features were extracted using Pyradiomics 2.1.0 (https://www.radiomics.io/pyradiomics.html). Eighteen first-order features and 61 texture features were extracted using each of the following: CE mask on CE-T1WI, CE mask on T2WI, and PT mask on T2WI. Fourteen shape features were extracted using each of the CE mask and PT mask. Thus, in total, 265 radiomic features were extracted. First-order features represent the distribution of gray values within an image, which are calculated from the histogram of voxel intensities. Shape features compute the three-dimensional size and shape of the ROI. Texture features describe relationships between neighboring voxels with similar or dissimilar values. We used 24 Gy level co-occurrence matrix, 16 Gy level run length matrix, 16 Gy level size zone matrix, and five neighboring gray tone difference matrix features for texture features. The details of the radiomic features are described elsewhere (https://www.radiomics.io/pyradiomics.html).

To assess the feature stability against perturbations in tumor segmentation, another radiologist (C.A. with 8 years of experience) drew ROIs on randomly sampled 60 patients using the same method, and ICC was calculated. Using the lower boundary of the 95% CI of the ICC value, feature stability was assigned to one of the following categories^41,42^: poor, lower boundary < 0.50; moderate, lower boundary ≥ 0.50 and < 0.75; good: lower boundary ≥ 0.75 and < 0.90; excellent, lower boundary ≥ 0.90.

### Model development

All machine learning analyses were performed using Python 3 with the Scikit-Learn library v0.21.2 and the Keras library v2.2.4 with TensorFlow used as the backend^43^.

#### Traditional machine learning combined with feature selection

**Figure 3:**
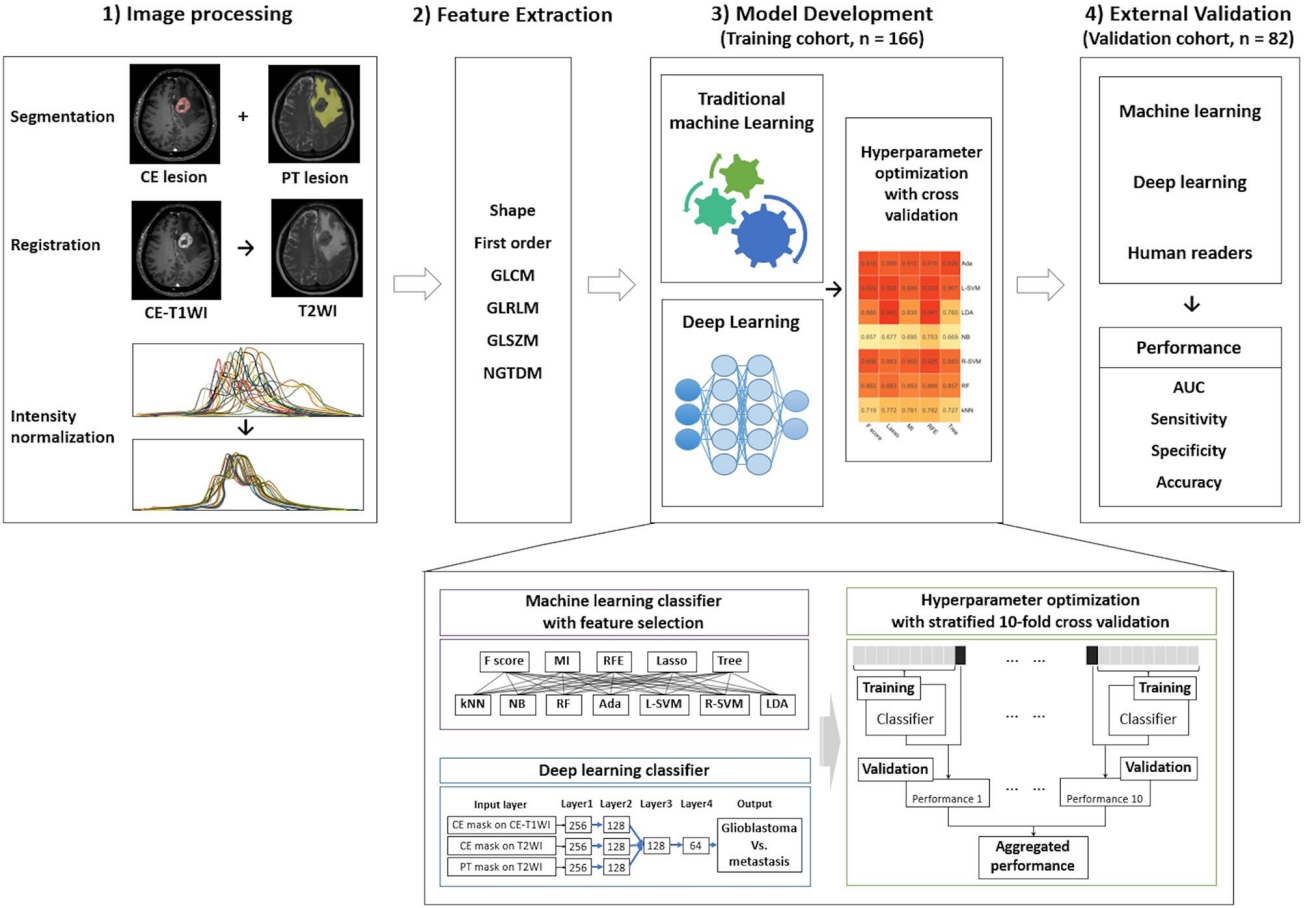
Pipeline of the study. *CE* contrast-enhancing, *PT* peritumoral T2 hyperintense, *CE-T1WI* contrast-enhanced T1-weighted image, *T2WI* T2-weighted image, *GLCM* gray level co-occurrence matrix, *GLRLM* gray level run length matrix, *GLSZM* gray level size zone matrix, *NGTDM* neighboring gray tone difference matrix, *MI* mutual information, *RFE* recursive feature elimination, *Lasso* least absolute shrinkage and selection operator, *kNN* k-nearest neighbor, *NB* naïve Bayes, *RF* random forest, *Ada* adaptive boosting, *L-SVM* linear support vector machine, *R-SVM* radial basis function support vector machine, *LDA* linear discriminant analysis, *AUC* area under the receiver operating characteristic curve.

In radiomics, a large number of features are extracted which form a high-dimensional feature space. The high dimensionality highly likely results in overfitting, undermining the generalizability of a prediction model (i.e., the “Curse of Dimensionality”)^44^. In addition, as the radiomic features are typically redundant, highly correlated features need to be reduced to avoid collinearity. Therefore, it is essential to reduce the dimension of the feature space by selecting the most relevant features. Hence, we used five methods for feature selection: univariate selection based on F score or mutual information (MI), recursive feature elimination (RFE), least absolute shrinkage and selection operator (LASSO), tree-based method. In combination with these feature-selection methods, seven machine learning classifiers were used to differentiate between glioblastoma and single brain metastasis: k-nearest neighbor, naïve Bayes, random forest, AdaBoost, L-SVM, SVM using radial basis function kernel, and LDA. Details of each feature selection and machine learning classifiers are summarized in Supplementary Table S7. For the 35 combinations of feature selection and classification methods, hyperparameters were optimized through stratified tenfold cross-validation in the training cohort (Fig. 3), and the best performing combination was identified based on the mean AUC. All analyses were repeated three times using (1) the CE mask, (2) the PT mask, and (3) combined mask data sets.

#### Feature importance

Feature importance was evaluated to examine how useful a radiomic feature was for a machine learning classifier to predict a target variable by calculating permutation feature importance with 100 repetitions^45^. Permutation feature importance is calculated by assessing the decrease in a model score when a single feature value is randomly shuffled; this procedure breaks the relationship between the feature and the target, thus the drop in the model score is indicative of how much the model depends on the feature. All measures of importance were scaled to have a maximum value of 100 and a minimum value of 0.

#### Deep learning

Multi-input DNN was implemented for deep learning. Our DNN had three independent input branches taking separately three data sets of radiomic features from the CE mask on CE-T1WI, CE mask on T2WI, and PT mask on T2WI (Supplementary Fig. S8). Each branch had two layers of fully connected dense layer followed by batch normalization and dropout with a rate of 0.5. The three branches were then merged into one common channel with three sequential layers of fully connected dense layer followed by batch normalization and a dropout rate of 0.5. Rectified linear unit activation function, He initialization, and an L2 regularizer were used in each layer, except for the final layer which performed binary classification using a sigmoid activation function. The RMSProp algorithm was used as an optimizer with a learning rate of 0.0002. Hyperparameters were optimized using stratified tenfold cross-validation in the training set, and the model with the highest mean AUC was chosen as the optimal model. All analyses were repeated three times, using (1) only the two CE mask input branches, (2) the PT mask branch alone, and (3) all the three input branches.

### Model validation

Each of the chosen optimal models was trained in the entire training cohort and validated in the validation cohort it had never seen. In the validation, AUC, sensitivity, specificity, and accuracy to distinguish glioblastoma from metastasis in the diagnosis of glioblastoma were calculated with 95% confidence intervals (CIs). The 95% CI of AUC was calculated by bootstrapping 10,000 times.

### Human reading

Two neuroradiologists (S.B. and S.S.A, with 3- and 10-years’ experience in neuro-oncological imaging, respectively) independently reviewed the images of the validation cohort. The readers were blinded to clinical information but were aware that the tumors were either glioblastoma or metastasis, without knowing the exact number of patients diagnosed with each entity. After anonymization and data randomization, the readers were given two image sets for each patient, which included T2WI and CE-T1WI images. The readers recorded a final diagnosis for each patient using a five-point scale: definite glioblastoma, likely glioblastoma, equivocal, likely metastasis, and definite metastasis. The diagnostic performance of the two human readers was assessed using R v3.3.2 (R Foundation for Statistical Computing, Vienna, Austria).

### Interrater agreement

The level of agreement was assessed using Fleiss’ kappa statistics between the classifying methods: the top three traditional machine learning models (i.e., AdaBoost, L-SVM, and LDA), DNN, and two human readers. The machine learning results with combined mask in the test set were used, as human readers assessed whole tumors on both T1 and T2 images in the test set. Kappa result was interpreted as follows: values of 0.01–0.20 as slight, 0.21–0.40 as fair, 0.41–0.60 as moderate, 0.61–0.80 as substantial, and 0.81–1.00 as almost perfect agreement.

## Supplementary information

Supplementary Information.
