# Supplementary material for: Robust performance of deep learning for distinguishing glioblastoma from single brain metastasis using radiomic features: model development and validation
Source: Sci Rep. 2020 Jul 21;10:12110. doi: 10.1038/s41598-020-68980-6 (PMC7374174; doi:10.1038/s41598-020-68980-6)
Supplement: Supplementary file 1 — Supplementary Information. [file 41598_2020_68980_MOESM1_ESM.pdf]

Supplementary information for:

Robust performance of deep learning for distinguishing glioblastoma from single brain metastasis using radiomic features: Model development and validation

Sohi Bae, M.D., Ph.D.[1+], Chansik An, M.D., Ph.D.[1,2+], Sung Soo Ahn, M.D., Ph.D.[3*], Hwiyoung Kim, Ph.D.[3*], Kyunghwa Han, Ph.D.[3], Sang Wook Kim[4], Ji Eun Park, M.D., Ph.D.[5], Ho Sung Kim, M.D., Ph.D.[5], Seung-Koo Lee, M.D., Ph.D.[3]

Contents

S1. Detailed diagnostic performance of different combinations of five feature-selection and seven classification methods, using CE mask

S2. Detailed diagnostic performance of different combinations of five feature-selection and seven classification methods, using PT mask

S3. Detailed diagnostic performance of different combinations of five feature-selection and seven classification methods, using combined mask

S4. Importance score of radiomics features used for seven machine learning classifiers

S5. Kappa statistics representing inter-rater agreement between various classifying methods

S6. Comparison of magnetic resonance imaging parameters of patients in the training cohort (from our institution) and those in the external validation cohort (from another tertiary medical center)

S7. Details of feature selection and classification methods

S8. Multi-input DNN implemented for deep learning

Supplementary Table S1. Detailed diagnostic performance of different combinations of five feature-selection and seven classification methods, using CE mask

Classifier	Feature selection method	Optimal feature number	Optimal hyperparameters	Mean AUC in the training set	AUC (95% CI) in the test set
kNN	F score	10	K = 2	0.690	0.744 (0.655, 0.829)
	MI	15	K =11	0.742	
	RFE	40	K = 8	0.737	
	Lasso	37	K = 13	0.707	
	Tree-based	150	K = 5	0.721	
NB	F score	130	NA	0.657	0.618 (0.513, 0.717)
	MI	20	NA	0.682	
	RFE	20	NA	0.676	
	Lasso	48	NA	0.675	
	Tree-based	63	NA	0.665	
RF	F score	10	N_estimators = 20, Min_samples leaf = 1	0.792	
	MI	10	N_estimators = 90, Min_samples leaf = 5	0.822	
	RFE	40	N_estimators = 140, Min_samples leaf = 3	0.839	0.773 (0.684, 0.857)
	Lasso	7	N_estimators = 10, Min_samples leaf = 3	0.837	
	Tree-based	73	N_estimators = 30, Min_samples leaf = 1	0.818	
AdaBoost	F score	160	N_estimators = 25, Learning_rate = 1	0.826	
	MI	100	N_estimators = 40, Learning_rate = 0.01	0.821	
	RFE	80	N_estimators = 35, Learning_rate = 0.5	0.860	
	Lasso	51	N_estimators = 40, Learning_rate = 1.5	0.870	0.858 (0.787, 0.926)
	Tree-based	68	N_estimators = 10, Learning_rate = 0.05	0.831	
L-SVM	F score	25	C = 0.01	0.837	
	MI	160	C = 0.05	0.837	
	RFE	15	C = 0.15	0.873	
	Lasso	53	C = 1	0.872	
	Tree-based	72	C = 0.1	0.875	0.833 (0.755, 0.904)
R-SVM	F score	65	C = 0.8, gamma = 0.0001	0.844	
	MI	30	C = 0.08, gamma = 0.0001	0.838	
	RFE	30	C = 0.1, gamma = 0.0005	0.860	
	Lasso	51	C = 0.02, gamma = 0.1	0.862	
	Tree-based	30	C = 2, gamma = 0.05	0.863	0.764 (0.674, 0.848)
LDA	F score	50	NA	0.723	
	MI	40	NA	0.792	
	RFE	40	NA	0.832	
	Lasso	37	NA	0.863	0.818 (0.737, 0.891)

	Tree-based	65	NA	0.813

CE = contrast-enhancing, AUC = area under the receiver operating characteristic curve, CI = confidence interval, , kNN = k-nearest neighbor, NB = naïve Bayes, RF = random forest, Ada = adaptive boosting, L-SVM = linear support vector machine, R-SVM = radial basis function support vector machine, LDA = linear discriminant analysis, MI = mutual information, RFE = recursive feature elimination, Lasso = least absolute shrinkage and selection operator

Supplementary Table S2. Detailed diagnostic performance of different combinations of five feature-selection and seven classification methods, using PT mask

Classifier	Feature selection method	Optimal feature number	Optimal hyperparameters	Mean AUC in the training set	AUC (95% CI) in the test set
kNN	F score	15	K = 2	0.701	
	MI	5	K = 8	0.699	
	RFE	50	K = 13	0.640	
	Lasso	6	K = 17	0.750	0.694 (0.589, 0.794)
	Tree-based	35	K = 3	0.660	
NB	F score	5	NA	0.673	0.753 (0.656, 0.838)
	MI	15	NA	0.645	
	RFE	25	NA	0.638	
	Lasso	6	NA	0.660	
	Tree-based	33	NA	0.658	
RF	F score	5	N_estimators = 10, Min_samples leaf = 4	0.639	
	MI	5	N_estimators = 20, Min_samples leaf = 4	0.739	0.700 (0.599, 0.798)
	RFE	5	N_estimators = 20, Min_samples leaf = 3	0.628	
	Lasso	6	N_estimators = 45, Min_samples leaf = 3	0.719	
	Tree-based	37	N_estimators = 30, Min_samples leaf = 2	0.723	
AdaBoost	F score	25	N_estimators = 10, Learning_rate = 1	0.790	
	MI	25	N_estimators = 25, Learning_rate = 2	0.746	
	RFE	15	N_estimators = 15, Learning_rate = 1	0.722	
	Lasso	38	N_estimators = 35, Learning_rate = 0.05	0.733	
	Tree-based	31	N_estimators = 20, Learning_rate = 2	0.816	0.761 (0.691, 0.827)
L-SVM	F score	25	C = 0.03	0.799	

	MI	25	C = 1	0.714	
	RFE	20	C = 0.008	0.830	0.803 (0.718, 0.879)
	Lasso	25	C = 0.2	0.816	
	Tree-based	34	C = 0.8	0.736	
	F score	60	C = 2, gamma = 0.005	0.782	
	MI	70	C =2, gamma = 0.006	0.783	
R-SVM	RFE	15	C = 0.001, gamma = 0.02	0.804	
	Lasso	25	C = 1, gamma = 0.01	0.817	0.753 (0.658, 0.839)
	Tree-based	34	C = 0.9, gamma = 0.01	0.754	
	F score	50	NA	0.761	
	MI	80	NA	0.818	0.768 (0.673, 0.856)
LDA	RFE	20	NA	0.763	
	Lasso	12	NA	0.798	
	Tree-based	33	NA	0.794	

PT = peritumoral T2 hyperintense, AUC = area under the receiver operating characteristic curve, CI = confidence interval, , kNN = k-nearest neighbor, NB = naïve Bayes, RF = random forest, Ada = adaptive boosting, L-SVM = linear support vector machine, R-SVM = radial basis function support vector machine, LDA = linear discriminant analysis, MI = mutual information, RFE = recursive feature elimination, Lasso = least absolute shrinkage and selection operator

Supplementary Table S3. Detailed diagnostic performance of different combinations of five feature-selection and seven classification methods, using combined mask

Classifier	Feature selection method	Optimal feature number	Optimal hyperparameters	Mean AUC in the training set	AUC (95% CI) in the test set
kNN	F score	25	K = 7	0.719	
	MI	20	K = 9	0.781	
	RFE	20	K = 6	0.782	0.738 (0.648, 0.822)
	Lasso	35	K = 6	0.772	
	Tree-based	97	K = 9	0.727	
NB	F score	25	NA	0.657	
	MI	20	NA	0.695	
	RFE	20	NA	0.753	0.713 (0.616, 0.804)
	Lasso	34	NA	0.677	
	Tree-based	100	NA	0.669	
RF	F score	20	N_estimators = 15, Min_samples leaf = 5	0.862	
	MI	120	N_estimators = 20, Min_samples leaf = 4	0.853	
	RFE	20	N_estimators = 25, Min_samples leaf = 2	0.866	
	Lasso	45	N_estimators = 45, Min_samples leaf = 4	0.883	0.780 (0.687, 0.862)
	Tree-based	96	N_estimators = 30, Min_samples leaf = 1	0.857	
AdaBoost	F score	250	N_estimators = 25, Learning_rate = 2	0.918	
	MI	65	N_estimators = 20 Learning_rate = 2	0.910	
	RFE	60	N_estimators = 20, Learning_rate = 0.5	0.910	
	Lasso	44	N_estimators = 35, Learning_rate = 0.1	0.899	
	Tree-based	96	N_estimators = 20, Learning_rate = 1	0.926	0.890 (0.823, 0.947)
L-SVM	F score	120	C = 0.1	0.929	

	MI	130	C = 0.4	0.899	
	RFE	70	C = 0.05	0.932	0.886 (0.822, 0.940)
	Lasso	34	C = 1.2	0.928	
	Tree-based	110	C = 1	0.901	
	F score	45	C = 2, gamma = 0.005	0.908	
	MI	20	C = 0.05, gamma = 0.04	0.900	
R-SVM	RFE	30	C = 3, gamma = 0.02	0.925	0.827 (0.749, 0.898)
	Lasso	8	C = 1, gamma = 0.06	0.883	
	Tree-based	250	C = 2, gamma = 0.002	0.883	
	F score	55	NA	0.860	
	MI	40	NA	0.830	
LDA	RFE	40	NA	0.941	
	Lasso	34	NA	0.945	0.883 (0.816, 0.941)
	Tree-based	98	NA	0.760	

AUC = area under the receiver operating characteristic curve, CI = confidence interval, , kNN = k-nearest neighbor, NB = naïve Bayes, RF = random forest, Ada = adaptive boosting, L-SVM = linear support vector machine, R-SVM = radial basis function support vector machine, LDA = linear discriminant analysis, MI = mutual information, RFE = recursive feature elimination, Lasso = least absolute shrinkage and selection operator

Supplementary Table S4. Importance score of radiomics features used for seven machine learning classifiers

Features	kNN	NB	RF	AdaBoost	L-SVM	R-SVM	LDA	Total
CE_mask_with_T2_image_firstorder_Median	1.00	0.42	0.32	0.16	1.00	1.00	0.46	4.35
CE_mask_with_T2_image_firstorder_10Percentile	NS	0.54	1.00	0.87	0.55	0.40	0.08	3.44
periT2_mask_with_T2_image_shape_Maximum2DDiameterRow	0.74	NS	NS	0.79	0.43	0.08	0.09	2.13
periT2_mask_with_T2_image_firstorder_Median	0.60	NS	NS	0.61	0.66	NS	NS	1.86
periT2_mask_with_T2_image_shape_Flatness	NS	NS	NS	0.61	0.34	0.76	0.05	1.76
periT2_mask_with_T2_image_glszm_GrayLevelNonUniformity	NS	NS	NS	0.34	0.22	0.84	0.16	1.56
periT2_mask_with_T2_image_glszm_SizeZoneNonUniformity	0.67	0.08	NS	0.34	0.07	0.24	0.00	1.40
CE_mask_with_T2_image_firstorder_InterquartileRange	0.77	0.38	NS	0.00	0.01	0.08	0.11	1.35
CE_mask_with_CE_image_glcm_Id	NS	NS	NS	0.32	0.01	NS	1.00	1.33
CE_mask_with_CE_image_glrlm_LowGrayLevelRunEmphasis	NS	NS	NS	0.00	0.45	0.20	0.63	1.28
CE_mask_with_T2_image_glszm_GrayLevelNonUniformity	NS	0.04	0.18	1.00	NS	NS	NS	1.22
CE_mask_with_T2_image_firstorder_Skewness	NS	0.04	0.59	0.47	NS	NS	NS	1.11
CE_mask_with_CE_image_firstorder_10Percentile	0.60	NS	0.27	0.00	0.00	0.16	NS	1.03
CE_mask_with_T2_image_firstorder_MeanAbsoluteDeviation	NS	1.00	NS	NS	NS	NS	NS	1.00
CE_mask_with_CE_image_glcm_InverseVariance	NS	NS	NS	0.00	0.37	0.04	0.58	0.98
CE_mask_with_CE_image_glcm_Autocorrelation	NS	NS	NS	0.00	0.00	NS	0.94	0.94
CE_mask_with_T2_image_firstorder_Mean	0.11	NS	0.14	0.00	0.03	0.44	0.20	0.91
CE_mask_with_CE_image_firstorder_Median	NS	NS	0.82	0.00	0.07	NS	NS	0.88
CE_mask_with_T2_image_glcm_DifferenceAverage	NS	NS	0.82	NS	NS	NS	NS	0.82
CE_mask_with_CE_image_glrlm_ShortRunLowGrayLevelEmphasis	NS	NS	NS	0.50	0.00	0.08	0.23	0.81
periT2_mask_with_T2_image_glrlm_RunVariance	NS	NS	0.77	NS	0.03	NS	NS	0.80
periT2_mask_with_T2_image_glrlm_LowGrayLevelRunEmphasis	NS	NS	0.77	NS	0.00	NS	NS	0.77
CE_mask_with_CE_image_glrlm_LongRunLowGrayLevelEmphasis	NS	NS	0.41	0.00	0.04	0.32	NS	0.77
periT2_mask_with_T2_image_firstorder_Skewness	NS	0.08	NS	0.68	0.00	NS	NS	0.77
CE_mask_with_CE_image_glszm_LowGrayLevelZoneEmphasis	NS	NS	0.45	0.00	0.03	0.16	0.07	0.71
CE_mask_with_CE_image_glcm_D	NS	NS	NS	0.00	NS	NS	0.71	0.71

ifferenceAverage								
CE_mask_with_T2_image_glcm_DifferenceEntropy	NS	NS	NS	0.45	0.04	NS	0.20	0.69
CE_mask_with_CE_image_shape_LeastAxisLength	0.18	0.13	0.36	0.00	NS	NS	NS	0.66
CE_mask_with_CE_image_firstorder_Mean	NS	NS	NS	0.50	0.00	0.16	NS	0.66
periT2_mask_with_T2_image_glrlm_ShortRunEmphasis	NS	NS	NS	0.58	NS	0.08	NS	0.66
periT2_mask_with_T2_image_firstorder_Minimum	0.33	0.00	0.32	0.00	NS	NS	NS	0.65
CE_mask_with_CE_image_glrlm_RunLengthNonUniformity	NS	NS	NS	NS	0.29	0.32	NS	0.61
CE_mask_with_CE_image_shape_Flatness	NS	NS	NS	0.58	NS	NS	NS	0.58
CE_mask_with_CE_image_shape_Maximum2DDiameterRow	0.12	0.17	NS	0.16	0.08	0.04	NS	0.57
CE_mask_with_CE_image_glszm_GrayLevelVariance	NS	NS	NS	0.45	0.11	NS	NS	0.55
CE_mask_with_T2_image_firstorder_RobustMeanAbsoluteDeviation	NS	NS	0.55	0.00	0.00	NS	NS	0.55
CE_mask_with_CE_image_glcm_SumAverage	NS	NS	NS	0.00	NS	0.08	0.46	0.54
CE_mask_with_CE_image_glcm_JointAverage	NS	NS	NS	0.00	0.07	NS	0.46	0.53
CE_mask_with_T2_image_firstorder_Minimum	0.53	NS	NS	NS	NS	NS	NS	0.53
CE_mask_with_T2_image_glcm_JointEnergy	NS	NS	0.36	0.00	0.08	0.08	NS	0.52
CE_mask_with_T2_image_firstorder_RootMeanSquared	NS	NS	NS	NS	NS	0.48	NS	0.48
CE_mask_with_CE_image_glszm_SmallAreaHighGrayLevelEmphasis	NS	NS	0.45	NS	NS	NS	NS	0.45
CE_mask_with_T2_image_glcm_MaximumProbability	NS	NS	0.18	0.00	0.11	0.08	0.07	0.44
CE_mask_with_CE_image_firstorder_InterquartileRange	NS	NS	NS	NS	0.14	0.28	NS	0.42
periT2_mask_with_T2_image_glrlm_RunEntropy	NS	NS	0.32	0.00	0.11	NS	NS	0.42
CE_mask_with_CE_image_firstorder_Minimum	0.37	0.00	0.05	NS	NS	NS	NS	0.41
periT2_mask_with_T2_image_shape_LeastAxisLength	0.40	0.00	NS	NS	NS	NS	NS	0.40
CE_mask_with_T2_image_ngtdm_Strength	NS	0.17	0.23	NS	NS	NS	NS	0.39
periT2_mask_with_T2_image_glcm_JointAverage	NS	NS	0.36	0.00	0.00	NS	0.02	0.39
CE_mask_with_CE_image_firstorder_90Percentile	0.37	NS	NS	0.00	0.00	NS	NS	0.37
CE_mask_with_T2_image_glrlm_LongRunEmphasis	NS	NS	0.36	NS	NS	NS	NS	0.36
CE_mask_with_T2_image_glcm_SumEntropy	NS	NS	0.36	NS	NS	NS	NS	0.36
CE_mask_with_CE_image_glrlm_ShortRunHighGrayLevelEmphasis	NS	NS	0.05	0.32	NS	NS	NS	0.36
CE_mask_with_CE_image_glcm_JointEnergy	NS	NS	NS	0.00	NS	0.36	NS	0.36

CE_mask_with_T2_image_glrlm_LongRunLowGrayLevelEmphasis	NS	NS	0.32	NS	NS	NS	NS	0.32
CE_mask_with_CE_image_glszm_HighGrayLevelZoneEmphasis	NS	NS	0.32	NS	NS	NS	NS	0.32
CE_mask_with_T2_image_shape_LeastAxisLength	0.18	0.13	0.00	NS	NS	NS	NS	0.30
CE_mask_with_T2_image_firstorder_Entropy	NS	NS	0.27	NS	0.03	NS	NS	0.30
periT2_mask_with_T2_image_glrlm_RunLengthNonUniformity	NS	NS	NS	0.00	0.00	0.28	NS	0.28
CE_mask_with_CE_image_firstorder_RobustMeanAbsoluteDeviation	NS	NS	NS	NS	NS	0.28	NS	0.28
CE_mask_with_T2_image_shape_Maximum2DDiameterRow	0.12	NS	NS	NS	0.08	NS	0.07	0.27
periT2_mask_with_T2_image_glszm_GrayLevelVariance	NS	NS	NS	0.00	0.25	NS	NS	0.25
CE_mask_with_T2_image_glszm_LowGrayLevelZoneEmphasis	NS	NS	0.23	NS	NS	NS	NS	0.23
periT2_mask_with_T2_image_glcm_Correlation	NS	NS	0.23	NS	NS	NS	NS	0.23
CE_mask_with_T2_image_glcm_JointAverage	NS	NS	0.23	0.00	NS	NS	NS	0.23
CE_mask_with_T2_image_glcm_JointEntropy	NS	NS	0.23	0.00	0.00	NS	NS	0.23
CE_mask_with_CE_image_ngtdm_Complexity	NS	NS	0.23	0.00	NS	NS	NS	0.23
CE_mask_with_CE_image_glrlm_RunVariance	NS	NS	0.23	NS	NS	NS	NS	0.23
CE_mask_with_CE_image_glcm_ClusterTendency	NS	NS	0.23	NS	NS	NS	NS	0.23
periT2_mask_with_T2_image_ngtdm_Complexity	0.21	NS	NS	NS	NS	NS	NS	0.21
CE_mask_with_T2_image_shape_Maximum2DDiameterSlice	NS	NS	NS	NS	NS	NS	0.21	0.21
CE_mask_with_T2_image_glcm_ClusterShade	NS	NS	NS	0.00	0.05	NS	0.13	0.19
CE_mask_with_CE_image_glcm_Contrast	NS	NS	0.18	NS	NS	NS	NS	0.18
CE_mask_with_CE_image_glrlm_RunPercentage	NS	NS	0.18	NS	NS	NS	NS	0.18
CE_mask_with_T2_image_glrlm_RunEntropy	NS	NS	0.18	NS	NS	NS	NS	0.18
CE_mask_with_T2_image_firstorder_Uniformity	NS	NS	0.18	0.00	NS	NS	NS	0.18
CE_mask_with_CE_image_glcm_JointEntropy	NS	NS	0.18	NS	NS	NS	NS	0.18
CE_mask_with_CE_image_firstorder_Entropy	NS	NS	0.18	NS	NS	NS	NS	0.18
CE_mask_with_CE_image_glcm_MaximumProbability	NS	NS	0.18	NS	NS	NS	NS	0.18
CE_mask_with_CE_image_firstorder_MeanAbsoluteDeviation	NS	NS	0.18	NS	NS	NS	NS	0.18
CE_mask_with_CE_image_glrlm_RunLengthNonUniformityNormalized	NS	NS	NS	0.00	0.17	NS	NS	0.17
CE_mask_with_T2_image_firstorder_Kurtosis	NS	0.17	NS	NS	NS	NS	NS	0.17

CE_mask_with_CE_image_glcm_SumEntropy	NS	NS	NS	0.00	0.16	NS	0.01	0.16
CE_mask_with_T2_image_glcm_InverseVariance	NS	NS	NS	NS	0.00	0.16	NS	0.16
CE_mask_with_T2_image_firstorder_Range	NS	NS	NS	NS	0.13	NS	NS	0.13
CE_mask_with_CE_image_glrlm_HighGrayLevelRunEmphasis	NS	NS	NS	0.00	0.00	NS	0.13	0.13
CE_mask_with_T2_image_glszm_HighGrayLevelZoneEmphasis	NS	NS	NS	0.00	NS	NS	0.13	0.13
CE_mask_with_CE_image_shape_MinorAxisLength	NS	0.13	NS	NS	NS	NS	NS	0.13
CE_mask_with_T2_image_glszm_SizeZoneNonUniformity	0.12	NS	NS	NS	NS	NS	NS	0.12
periT2_mask_with_T2_image_shape_Maximum3DDiameter	NS	NS	NS	0.00	0.08	0.04	NS	0.12
CE_mask_with_CE_image_firstorder_Maximum	NS	NS	NS	0.00	0.12	NS	NS	0.12
periT2_mask_with_T2_image_firstorder_MeanAbsoluteDeviation	0.11	0.00	NS	NS	NS	NS	NS	0.11
periT2_mask_with_T2_image_glszm_GrayLevelNonUniformityNormalized	NS	NS	NS	0.00	0.09	NS	NS	0.09
CE_mask_with_T2_image_glrlm_ShortRunEmphasis	NS	NS	0.09	NS	NS	NS	NS	0.09
periT2_mask_with_T2_image_shape_VoxelVolume	NS	NS	NS	0.00	0.05	0.00	0.04	0.09
CE_mask_with_T2_image_glcm_Id	NS	NS	NS	0.00	NS	NS	0.09	0.09
periT2_mask_with_T2_image_ngtdm_Strength	0.00	0.08	NS	0.00	NS	NS	NS	0.08
CE_mask_with_T2_image_firstorder_Energy	NS	NS	NS	NS	0.00	NS	0.07	0.07
periT2_mask_with_T2_image_shape_MeshVolume	NS	NS	NS	0.00	0.07	0.00	NS	0.07
CE_mask_with_T2_image_glszm_SizeZoneNonUniformityNormalized	NS	NS	NS	0.00	0.07	NS	NS	0.07
CE_mask_with_CE_image_glcm_DifferenceEntropy	NS	NS	NS	0.00	0.00	NS	0.05	0.05
CE_mask_with_T2_image_glcm_SumSquares	NS	NS	NS	NS	0.04	NS	NS	0.04
CE_mask_with_CE_image_firstorder_RootMeanSquared	NS	NS	NS	0.00	0.03	NS	0.01	0.03
CE_mask_with_CE_image_shape_SurfaceVolumeRatio	NS	NS	NS	0.00	0.00	NS	0.03	0.03
CE_mask_with_T2_image_shape_SurfaceVolumeRatio	NS	NS	NS	0.00	NS	NS	0.03	0.03
CE_mask_with_CE_image_firstorder_Skewness	NS	NS	NS	0.00	0.03	NS	NS	0.03
periT2_mask_with_T2_image_glszm_ZoneEntropy	NS	NS	NS	NS	0.03	NS	NS	0.03
periT2_mask_with_T2_image_glcm_SumAverage	NS	NS	NS	0.00	0.00	NS	0.02	0.02
CE_mask_with_T2_image_shape_Elongation	NS	NS	NS	NS	0.01	NS	NS	0.01
CE_mask_with_T2_image_glcm_Idm	NS	NS	NS	0.00	0.01	NS	NS	0.01
periT2_mask_with_T2_image_glrl	NS	NS	NS	0.00	0.01	NS	NS	0.01

m_GrayLevelNonUniformity								
periT2_mask_with_T2_image_firstorder_90Percentile	NS	NS	NS	NS	0.01	NS	NS	0.01

Features with zero total score were not included.

kNN = k-nearest neighbor, NB = naïve Bayes, RF = random forest, ADA = adaptive boosting, L-SVM = linear support vector machine, R-SVM = radial basis function support vector machine, LDA = linear discriminant analysis, CE = contrast-enhancing, NS = not selected for this classifier.

Supplementary Table S5. Kappa statistics representing inter-rater agreement between various classifying methods

	**AdaBoost**	**L-SVM**	**LDA**	**DNN**	**Human 1**	**Human 2**
**AdaBoost**	1.0	0.607	0.644	0.742	0.17	0.421
**L-SVM**	0.607	1.0	0.631	0.685	0.238	0.466
**LDA**	0.644	0.631	1.0	0.663	0.285	0.546
**DNN**	0.742	0.685	0.663	1.0	0.311	0.479
**Human 1**	0.17	0.238	0.285	0.311	1.0	0.505
**Human 2**	0.421	0.466	0.546	0.479	0.505	1.0

AdaBoost = adaptive boosting, L-SVM = linear support vector machine, R-SVM = radial basis function support vector machine, LDA = linear discriminant analysis.

Supplementary Table S6. Comparison of magnetic resonance imaging parameters of patients in the training cohort (from our institution) and those in the external validation cohort (from another tertiary medical center).

Pulse Sequence	Our institution	External validation set
T2-weighted image		
Repetition Time (ms)	3000–9000	3000
Echo Time (ms)	80–120	80–120
Matrix	256 × 256	256 × 256
Section thickness (mm)	5	4–7
Field of view	220–240	230–240
Contrast-enhanced T1-weighted image		
Repetition Time (ms)	6.3–8.3	6.4–10.1
Echo Time (ms)	2.9–4	2.8–4.8
Matrix	256 × 256 or 192 × 192	512 × 512
Section thickness (mm)	1	0.5
Field of view	224–240	224–230

Supplementary Table S7. Details of feature selection and classification method

	Acronym	Feature selection method
1	F score	F score
2	MI	Mutual information
3	RFE	Recursive feature elimination
4	LASSO	Least absolute shrinkage and selection operator
5	Tree-based	Tree-based

	Acronym	Classification method
1	kNN	k-nearest neighbor
2	NB	Naïve Bayes
3	RF	Random forest
4	AdaBoost	Adaptive boosting
5	L-SVM	Support vector machine using linear kernel
6	R-SVM	Support vector machine using radial basis function kernel
7	LDA	Linear discriminant analysis

Feature selection methods

1. F score[1]

The F score is a univariate feature selection method. It is based on F-test estimate the degree of linear dependency between two random variables. It is to find a subset of features, such that in the data space spanned by the selected features, the distances between data points in different classes are as large as possible, while the distances between data points in the same class are as small as possible.

2. MI (Mutual information)[2]

The MI method measures arbitrary dependencies between random variables. It is suitable for assessing the information content of features in complex classification tasks, where methods based on linear relations are prone to mistakes

3. RFE (Recursive feature elimination)[3]

The RFE selects features by recursively considering smaller and smaller sets of features. The estimator is trained on an initial set of features and weights are assigned to each. Then the features whose absolute weights are the smallest are eliminated from the current features in a backwards elimination manner. This procedure is recursively repeated until the desired number of features is reached

4. LASSO (Least absolute shrinkage and selection operator)[4]

The LASSO performs two tasks: regularization and feature selection. The LASSO minimizes the residual sum of squares subject to the sum of the absolute value of the coefficients being less than a constant. Because of the nature of this constraint it tends to produce some coefficients that are exactly 0 and hence gives interpretable models.

5. Tree-based[5]

The tree-based algorithm utilizes a tree structure to model relationships among the features and the potential outcomes. A standard CART algorithm is used to select the split predictor that maximizes the split-criterion gain over all possible splits of all predictors. Finding the optimal size of the tree helps improve predictive accuracy through the reduction of overfitting.

Classification methods

1. kNN (k-nearest neighbor)[6]

The kNN algorithm assigns to unclassified observation (incoming test sample) the class/category/label of the nearest sample (using metric) in training set. The letter k is a variable term implying that any number of nearest neighbors could be used.

2. NB (Naïve Bayes)[7]

The NB model assumes that observations have a multivariate distribution, given class membership, but the predictor and features composing the observation are independent.

3. RF (Random forest)[8]

The RF model uses ensembles of trees, where each tree in the ensemble is grown in accordance with a random parameter. Final predictions are obtained by aggregating over the ensemble. As the base constituents of the ensemble are tree-structured predictors, and since each of these trees is constructed using an injection of randomness.

4. AdaBoost (Adaptive boosting)[9]

The ADA is a general method for generating a strong classifier out of a set of weak classifiers. It works even when the classifiers come from a continuum of potential classifiers (such as neural networks, linear discriminants, etc.).

5. L-SVM (Support vector machine using linear kernel)[10]

The L-SVM model constructs a hyperplane separating data into two classes. The optimal hyperplane maximizes a margin surrounding itself, which creates boundaries for positive and negative classes.

6. R-SVM (Support vector machine using radial basis function kernel)[10]

The R-SVM is a nonlinear version of L-SVM with Gaussian kernel function that projects original features onto a higher dimensional space via a nonlinear mapping function where it becomes linearly separable.

7. LDA (Linear discriminant analysis)[11]

The LDA model searches for the vectors in the underlying space that best discriminate among classes (rather than those that best describe the data). Given a number of independent features relative to which the data is described, LDA creates a linear combination of these which yields the largest mean differences between the desired classes. The goal is to maximize the between-class measure while minimizing the within-class measure.

Supplementary Figure S8. Multi-input DNN implemented for deep learning

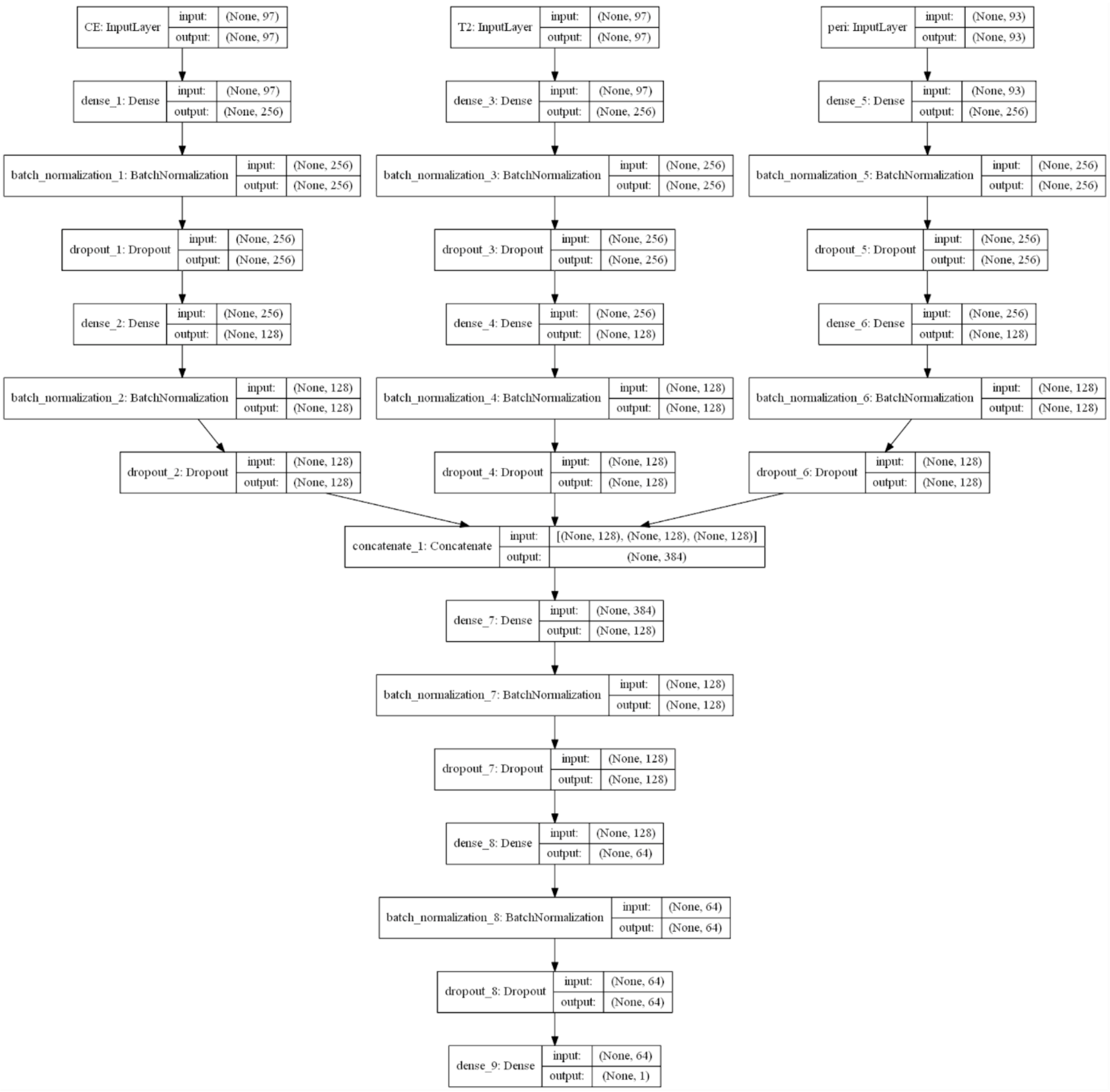

References

1. Gu, Q., Li, Z. & Han, J. Generalized fisher score for feature selection. *arXiv preprint arXiv:1202.3725* (2012).
2. Battiti, R. Using mutual information for selecting features in supervised neural net learning. *IEEE Transactions on neural networks* **5**, 537-550 (1994).
3. Guyon, I., Weston, J., Barnhill, S. & Vapnik, V. Gene selection for cancer classification using support vector machines. *Machine learning* **46**, 389-422 (2002).
4. Tibshirani, R. Regression shrinkage and selection via the lasso. *Journal of the Royal Statistical Society: Series B (Methodological)* **58**, 267-288 (1996).
5. Loh, W. Y. Classification and regression trees. *Wiley Interdisciplinary Reviews: Data Mining and Knowledge Discovery* **1**, 14-23 (2011).
6. Cover, T. & Hart, P. Nearest neighbor pattern classification. *IEEE transactions on information theory* **13**, 21-27 (1967).
7. Friedman, J., Hastie, T. & Tibshirani, R. *The elements of statistical learning*. Vol. 1 (Springer series in statistics New York, 2001).
8. Biau, G. Analysis of a random forests model. *Journal of Machine Learning Research* **13**, 1063-1095 (2012).
9. Rojas, R. AdaBoost and the super bowl of classifiers a tutorial introduction to adaptive boosting. *Freie University, Berlin, Tech. Rep* (2009).
10. Cristianini, N. & Shawe-Taylor, J. *An introduction to support vector machines and other kernel-based learning methods*. (Cambridge university press, 2000).
11. Martínez, A. M. & Kak, A. C. Pca versus lda. *IEEE transactions on pattern analysis and machine intelligence* **23**, 228-233 (2001).
